# ATPase activity of human binding immunoglobulin protein (BiP) variants is enhanced by signal sequence and physiological concentrations of Mn^2+^

**DOI:** 10.1002/2211-5463.12645

**Published:** 2019-07-10

**Authors:** Sravanthi Bandla, Suraya Diaz, Heinz Peter Nasheuer, Una FitzGerald

**Affiliations:** ^1^ School of Natural Sciences, National University of Ireland Galway Galway Ireland; ^2^ Galway Neuroscience Centre, National University of Ireland Galway Galway Ireland; ^3^ Centre for Chromosome Biology National University of Ireland Galway Galway Ireland

**Keywords:** ATPase, B‐cell immunoglobulin binding protein, endoplasmic reticulum chaperone, endoplasmic reticulum retention sequence KDEL, Glucose‐regulated protein 78 kDa, signal sequence

## Abstract

B‐cell immunoglobulin binding protein (BiP) is an essential endoplasmic reticulum (ER) chaperone normally found in the ER lumen. However, BiP also has other extracellular and intracellular functions. As it is unclear whether peripheral BiP has a signal and/or ER retention sequence, here we produced and biochemically characterised four variants of BiP. The variants differed depending on the presence or the absence of signal and ER retention peptides. Proteins were purified using nickel affinity chromatography, and variant size and quality were confirmed using SDS/PAGE gels. The thermal denaturing temperature of these proteins was found to be 46–47 °C. In addition, we characterised nucleotide binding properties in the absence and the presence of divalent cations. Interestingly, in the absence of cations, ADP has a higher binding affinity to BiP than ATP. The presence of divalent cations results in a decrease of the *K*_d_ values of both ADP and ATP, indicating higher affinities of both nucleotides for BiP. ATPase assays were carried out to study the enzyme activity of these variants and to characterise the kinetic parameters of BiP variants. Variants with the signal sequence had higher specific activities than those without. Both Mg^2+^ and Mn^2+^ efficiently stimulated the ATPase activity of these variants at low micromolar concentrations, whereas calcium failed to stimulate BiP ATPase. Our novel findings indicate the potential functionality of BiP variants that retain a signal sequence, and also reveal the effect of physiological concentrations of cations on the nucleotide binding properties and enzyme activities of all variants.

**Abbreviations**
BiP: B‐cell immunoglobulin binding protein
DSF: differential scanning fluorimetry
ER: endoplasmic reticulum
 glucose‐regulated protein 78 kDa
HSP70: heat shock protein 70 kDa
HuBiP: human BiP
 inositol‐requiring enzyme
 multiple sclerosis
 Protein Kinase RNA‐like Endoplasmic Reticulum Kinase
RAMP: resolution‐associated molecular pattern
RA: rheumatoid arthritis

B‐cell immunoglobulin binding protein (BiP), a heat shock protein 70 protein family member, is an indispensable endoplasmic reticulum (ER) chaperone normally present within the ER lumen 1. Classically, BiP functions to assist in protein folding, prevent the aggregation of intermediates and aid calcium binding as well as the trafficking of misfolded protein to the ER‐associated degradation system 1, 2. BiP is comprised of multiple functional sequences (Fig. 1A). The N‐terminal signal sequence of human BiP (huBiP, amino acids 1–18) directs the protein to the ER, whereas the C‐terminal KDEL sequence (amino acids 651–654) functions to retain BiP within the ER 3. The nucleotide binding domain (NBD) (amino acids 125–280) in the N terminus binds to ATP and ADP in a cycle that controls protein substrate binding and release, while the substrate binding domain (SBD) in the C terminus (amino acids 420–500) aids the interaction with substrate protein chains. In addition, a hydrophobic flexible linker region (amino acids 409–419) mediates BiP‐BiP interactions and is exposed when BiP is in the ADP‐bound state, but is not accessible when BiP is in the ATP‐bound state 4, 5, 6. Cations act as cofactors in nucleotide binding and Mg^2+^ is the chosen cation for BiP nucleotide binding studies, although Mn^2+^ and Ca^2+^ may also affect ATPase activity 7, 8.

**Figure 1:**
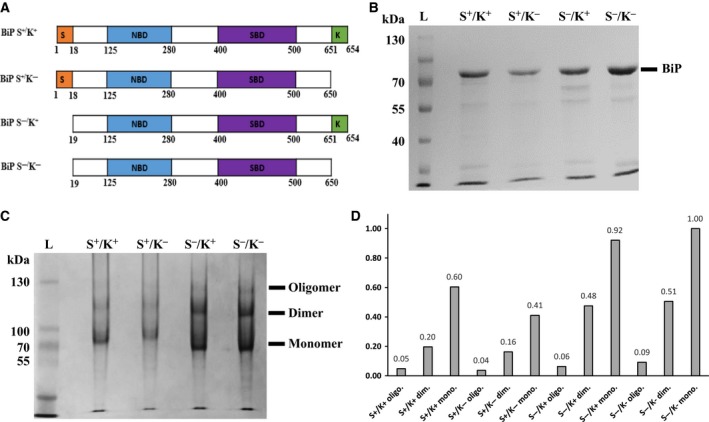
Structure, cloning and visualisation of full‐length huBiP and variants. (A) Schematic illustration of recombinant human full‐length BiP and its variants. Signal sequence (orange; S), NBD (blue), SBD (violet), KDEL sequence (green; K). Amino acid numbering is based on information contained in Wang *et al.,* 2017 and Zhang *et al.,* 2010 1, 23. (B) SDS/PAGE of purified recombinant full‐length huBiP and variant BiP proteins. Proteins were expressed in BL21–CodonPlus (DE3)‐RIL cells and purified using metal chelate chromatography as described in the Materials and methods part. A 10% SDS‐polyacrylamide gel was used to separate the denatured protein. The Coomassie Brilliant Blue‐stained gel is presented. On each lane of the SDS gel, 15 μg of either purified full‐length BiP or variant proteins was loaded as indicated. (C) A representative image of native PAGE gel of purified full‐length BiP and variants. Fifteen microgram of purified full‐length BiP and variant proteins was loaded on each lane of a 6% polyacrylamide gel, and native PAGE was performed using Tris‐Glycine as running buffer. After the electrophoresis, the gel was stained with Coomassie Brilliant Blue. (D) The quantification of the different protein species in panel C was carried out as described in the Materials and methods section. In diagram, the area under the absorption curve of the monomeric (mono.) BiP variant S^−^/K^−^ (last lane in panel C) was arbitrarily set to 1; dimeric (dim.) and oligomeric (oligo.) forms of each variant protein are presented as relative values.

Despite the presence of signal and KDEL sequences, BiP has also been detected in/at the nucleus, mitochondria, on the cell surface and extracellularly 9, and *in vitro*, BiP release into the cell culture medium of oviductal epithelial cells has been reported 10. In humans, BiP has also been found in the synovial fluid and its precursor was found in the saliva of individuals with Rheumatic arthritis (RA) 12. Tsunemi *et al*. 11 also detected BiP in the sera of gastric cancer patients. In circulation, BiP may behave as a resolution‐associated molecular pattern (RAMP), by antagonising proinflammatory mediators and re‐establishing homeostasis of the immune system 13. These properties led to clinical trials of recombinant BiP in therapies for RA 12, 14, 15.

It is possible that malfunctioning protein trafficking or an aberrant ER retention process could lead to extracellular distribution of BiP variants such as BiP with or without its signal and/or retention sequence. Therefore, we hypothesised that BiP variants could execute non‐ER‐associated physiological functions intra‐ or extracellularly, dictated in part by the presence or absence of signal or retention sequences. To test potential differences in the biochemical properties of huBiP variants, we completed an extensive characterisation of their stability and nucleotide binding in the presence of different cations. From these studies, we conclude that the presence of the signal sequence influences the nucleotide binding and ATPase activity of these proteins in the presence and absence of divalent cations.

## Materials and methods

### Cloning of full‐length human BiP and variants

Human S^−^/K^+^ BiP cDNA 16 was kindly provided by K. Polizzi of Imperial College, London, UK (amino acids 20–654 from gene accession number NM_005347) and was used as a template in PCR mixtures to generate different variants of BiP (full‐length BiP S^+^/K^+^, having both the signal sequence and the ER retention KDEL, BiP S^+^/K^−^, containing the signal sequence but lacking the C‐terminal KDEL peptide, BiP S^−^/K^+^, without signal sequence and having the KDEL peptide, and BiP S^−^/K^−^, lacking both the signal sequence and the KDEL peptide; Fig. 1A). Two forward and two reverse primers were used: one forward primer with signal sequence 5′‐CAGGATCCACATATGAAGCTCTCCCTGGTGGCCGCGATGCTGCTGCTGCTCAGCGCGGCGCGGGCCGAGGAGGAGGACAAGAAGGAGGACGTG‐3′ (to amplify full‐length BiP (BiP S^+^/K^+^) and BiP S^+^/K^−^) and one forward primer without signal sequence 5′‐CAGGATCCACATATGGAGGAGGAGGACAAGAAGGAGGACGTG‐3′ (to amplify BiP S^−^/K^+^ and BiP S^−^/K^−^); one reverse primer with KDEL sequence 5′‐GGTCTCGAGCAACTCATCTTTTTCTGCTGTATCCTCTTCACCAGTTGG‐3′ (amplifying BiP S^+^/K^+^ and BiP S^−^/K^+^) and the other reverse primer without KDEL sequence 5′‐GGTCTCGAGTTCTGCTGTATCCTCTTCACCAGTTGG‐3′ (amplifying BiP S^+^/K^−^ and BiP S^−^/K^−^). The amplified DNA sequences were ligated into pGEM T easy vector (Promega, Madison, Wisconsin, USA), sequenced, restriction digested using NdeI and XhoI restriction enzyme and ligated into the pET22b vector (Merck, County Cork, Ireland). Cloned sequences were sequenced by Eurofin genomics (Eurofin Genomics, Ebersberg, Germany) to confirm the absence of mutations that could be introduced during PCR.

### Protein expression and purification

The pET22b constructs encoding human full‐length BiP and BiP variants with a 5xHis‐tag at their C termini were introduced into *Escherichia coli* BL21‐CodonPlus (DE3)‐RIL competent cells (Agilent Technologies, Edinburgh, UK) for protein expression. A single colony from each protein was grown overnight at 180 r.p.m. at 37 °C supplemented with ampicillin (100 μg·mL^−1^) (Melford, Ipswich, UK) and chloramphenicol (25 μg·mL^−1^) (Sigma‐Aldrich, Dublin, Ireland) and grown at 180 r.p.m. and at 37 °C. After induction at an OD_600_ of 0.5 with 1 mm IPTG (Melford), BiP S^+^/K^+^ and BiP S^+^/K^−^ expressing *E. coli* were incubated for 3 h at 26 °C and 180 r.p.m. BiP S^−^/K^+^ and BiP S^−^/K^−^ were induced with 1 mm IPTG, and cells were grown for 3 h at 180 r.p.m. and 37 °C. The cells were harvested at 350 ***g*** for 5 min at 4 °C and lysed with 30 mm HEPES‐KOH, pH 7.8, 150 mm NaCl, 0.5% NP‐40 (v/v) (Honeywell Fluka Chemicals, Fisher Scientific, Dublin, Ireland), 1× EDTA‐free protease inhibitors and 1 mg·mL^−1^ lysozyme (Sigma‐Aldrich) (buffer A) for 3 h at 4 °C. The lysates were subsequently incubated for 1 h with 1 mg·mL^−1^ DNase I (Sigma‐Aldrich) at 4 °C and centrifuged for 30 min at 27 000 ***g*** at 4 °C. The supernatant was added to a pre‐equilibrated HIS‐Select® Nickel Affinity Gel (Sigma‐Aldrich) in 30 mm HEPES‐KOH, pH 7.8, 150 mm NaCl (buffer B) and incubated for 10 min at 4 °C. Afterwards, the resin was washed with buffer B and the different proteins eluted using 30 mm HEPES‐KOH, pH 7.8, 150 mm NaCl and 250 mm imidazole (buffer C). The eluted proteins were concentrated using Vivaspin concentrators (5 kDa molecular weight cut‐off; Sartorius, Göttingen, Germany) and the buffer exchanged to buffer B 10 desalting columns (GE Health Care, Cardiff, UK) according to the manufacturer's instructions. Protein concentrations were determined using a NanoDrop 2000 Spectrophotometer (Thermo Fisher Scientific, Dublin, Ireland) with 250 to 300 nm spectra collection and a reading at 280 nm.

### SDS/PAGE

Reducing SDS/PAGE 6× sample loading buffer was added to purified proteins with subsequent denaturation for 10 min at 95 °C as previously described 17. The samples were loaded onto 10% acrylamide gel and run using 1× Tris‐Glycine buffer (25 mm Tris, 0.192 M glycine, 0.1% SDS) at 200 V for 1 h at room temperature until the PageRuler™ prestained protein ladder (Thermo Fisher Scientific) was fully resolved. The gels were stained with a solution containing 2.5 g of Coomassie Brilliant Blue R‐250 in 450 mL methanol, 100 mL glacial acetic acid (all from Sigma‐Aldrich) and 450 mL milliQ water.

The expression of huBiP variants in crude cell extracts and the purified recombinant proteins was verified by western blotting using a polyclonal rabbit anti‐BiP‐antibody (Abcam, Cambridge, UK, Cat # Ab32618, RRID:AB_732737, data not shown).

### Native PAGE

Native PAGE was carried out as previously described with slight modifications 18. In short, nonreducing 6× sample loading buffer (600 mm Tris/HCl pH7.8, 50% glycerol, 0.02% bromophenol blue) was mixed with 15 μg of recombinant protein and loaded onto 6% gel. The gel was run in Tris‐Glycine buffer without SDS (25 mm Tris and 192 mm glycine) at 200 V for 45 min until the PageRuler™ prestained protein ladder (Thermo Fisher Scientific) was fully resolved and stained with Coomassie Brilliant Blue R‐250 as described above.

### ATPase activity measurements

ATPase assays were performed using a Malachite Green Phosphate Assay Kit (Universal Biologicals Ltd, Cambridge, UK) according to the manufacturer's instructions. Reaction mixtures were prepared in triplicate in a final volume of 160 μL, using 10 μg of protein in 30 mm HEPES‐KOH, pH 7.8, 150 mm NaCl, 20 μm ATP and 2 mm MgCl_2_ and incubated for 60 min at 37 °C (adapted from 19). The samples were transferred into 96‐well plates, and the concentration of phosphate was measured at 620 nm using a Varioskan flash plate reader (Thermo Fisher Scientific) with skanit software 2.4.3 (Fisher Scientific, Dublin, Ireland). For calculating the kinetic parameters such as *K*_M_ and *V*_max_, the same protocol was used except that the concentrations of cations and nucleotides were varied. The resulting data were analysed, and kinetic parameters calculated using the Michaelis‐Menten equation with graphpad prism 5 software (GraphPad Software, San Diego, CA, USA).

### Differential scanning fluorimetry

Differential scanning fluorimetry (DSF) was used to study the stability, nucleotide binding and the effect of divalent cations on the nucleotide binding, as described elsewhere 20, 21. Experiments were performed using 1× fluorescent dye Sypro Orange (Applied Biosystems, Dublin, Ireland) in 30 mm HEPES‐KOH, pH 7.8, 150 mm NaCl, with full‐length huBiP and variants, set at a fixed concentration of 1 μm alone, or with titrations of cofactors (Table 1). The samples were transferred to 96‐well Fast Thermal Cycling Plates (Applied Biosystems, Thermo Fisher Scientific) and subjected to a heating ramp of 1.0 °C per min from 25 to 90 °C, with fluorescence intensity readings at 470 and 550 nm of excitation and emission wavelengths, respectively, using a StepOnePlus System (Applied Biosystems). Data were analysed using protein thermal shift software v1.1 (Applied Biosystems) and graphpad prism 5 software (GraphPad) as described previously 20, 21. The dissociation constant was calculated according to Vivoli *et al*. 21 using the following equations:

(1) $$K_{d}=c^{\theta }e^{\Delta_{\gamma }G/\mathrm{RT}}$$

(2) ΔG=ΔH-TΔS

**Table 1:** BiP nucleotide binding activity is modulated by the presence of divalent cations

BiP variant	Nucleotide bound	Cation		*K*_d_ [μm]	Standard deviation [μm]
S^+^/K^+^	ATP	None		766	157
		MgCl_2_	50 μm	14	2
			2 mm	44	5
		MnCl_2_	25 μm	12	2
			2 mm	27	3
		CaCl_2_	2 mm	30	4
	ADP	None		138	16
		MgCl_2_	50 μm	100	18
			2 mm	59	7
		MnCl_2_	25 μm	13	2
			2 mm	21	2
		CaCl_2_	2 mm	19	2
S^−^/K^−^	ATP	None		352	75
		MgCl_2_	50 μm	10	2
			2 mm	13	3
		MnCl_2_	25 μm	2	0.5
			2 mm	5	3
		CaCl_2_	2 mm	10	3
	ADP	None		107	17
		MgCl_2_	50 μm	77	9
			2 mm	27	6
		MnCl_2_	25 μm	15	2
			2 mm	15	4
		CaCl_2_	2 mm	5	0.48
S^+^/K^−^	ATP	None		610	233
	ADP	None		188	15
S^−^/K^**+**^	ATP	None		945	262
	ADP	None		98	11

In these equations, *c* ^θ^ is the standard reference concentration, Δ_γ_ *G* is the Gibbs free energy change of the reaction, *R* is the molar gas constant, Δ*H* is the enthalpy change in the reaction, and Δ*S* is the entropy change in the reaction.

### Protein quantification

The molecular weights and molar extinction coefficient of the proteins, 30 495 M^−1 ^cm^−1^ at 280 nm, were calculated using Expasy Protparam tool (https://web.expasy.org/protparam/) and the human amino acid sequence. To determine the concentration, the absorption of purified proteins was measured at 280 nm using a NanoDrop 2000 Spectrophotometer. The relative amounts of oligomeric species of the BiP variants in Coomassie‐stained native polyacrylamide gels were quantified by scanning the native PAGE gel using an Epson Dual Lens system (model Epson perfection V700 photo; Digital Ice Technologies, Hemel Hempstead, UK), and band intensities were quantified using the imagej software (NIH, Bethesda, MD, USA). All values were expressed relative to monomeric S^−^/K^−^.

### Statistical analysis

Statistical analysis was carried out using an unpaired *t*‐test. A minimum of two experiments were performed in triplicate and data depicted as SD ± mean.

## Results

### Human BiP expression, purification and preliminary characterisation

Various forms of BiP have been described to occur under physiological and pathological conditions including ER stress and on the cell surface of cancer cells 9, 22, 23, 24. To investigate biochemical activities of these BiP variants, we cloned huBiP variants containing or lacking the signal and KDEL sequence (Fig. 1) and produced them in BL21 codon plus RIL *E. coli* cells and have a C‐terminal 6× His‐tag, enabling purification using immobilised metal affinity columns (Fig. 1A,B). Here, we demonstrated the feasibility of producing, from 500 mL cultures of *E. coli* BL21‐CodonPlus (DE3)‐RIL cells, 5–8 mg of full‐length huBiP and its variants, at a concentration of 10–20 μg·μL^−1^. Batch quality was confirmed using SDS/PAGE. Coomassie Brilliant Blue staining of gels showed that the size of the purified proteins was larger than 70 kDa, as expected (Fig. 1B). The SDS/PAGE also showed that the purified proteins were highly pure and had minimal contaminants or degradation. The estimated molecular weights of the proteins, derived from the cDNA sequences, were as follows: S^+^/K^+^, 73.4 kDa; S^+^/K^−^, 72.9 kDa; S^−^/K^+^, 71.7 kDa; S^−^/K^−^, 71.2 kDa. Thus, the maximal difference between variants is about 2 kDa, an amount that is not detectable using SDS/PAGE in a protein of this size.

**Figure 2:**
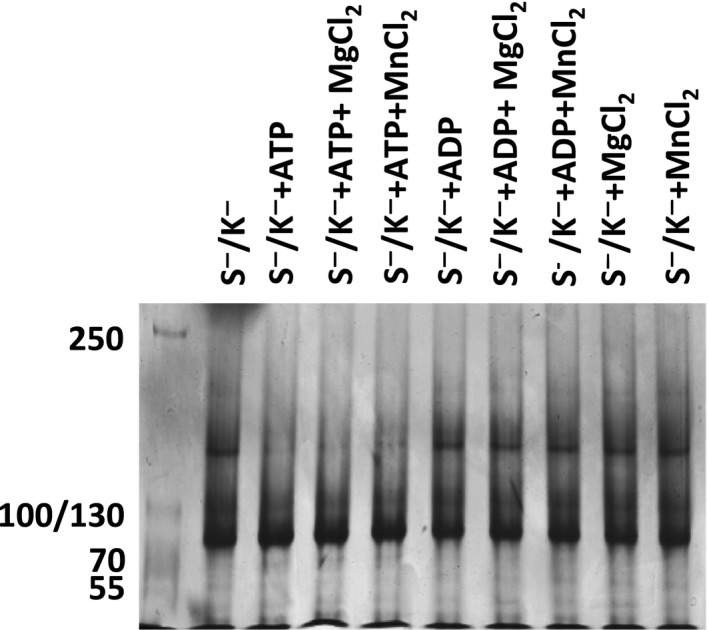
Effect of nucleotides and cations on BiP. BiP S^−^/K^−^ (20 μg) in the presence of 2 mm of Mg^2+^ or Mn^2+^ or 1 mm of ATP or ADP and a combination of cations and nucleotides as indicated were incubated at 25 °C for 10 min and were loaded on a Bis‐Tris 6% gel, and native PAGE was performed using Tris‐Glycine as running buffer. After electrophoresis, the gel was then stained with Coomassie Brilliant Blue.

To confirm the oligomeric state of the recombinant proteins, samples were separated on a native PAGE gel. All variants were found to exist as monomers, dimers and higher oligomer forms (Fig. 1C). For all variants, about 5% of native protein was oligomeric, while between 20% and 30% was dimeric, and 60–70% monomeric protein (Fig. 1D). We also used the nondenaturing PAGE to study the effect of divalent cations and nucleotides on oligomerisation. In the presence of ATP alone or in combination with divalent cations, dimers were converted into monomers, whereas cations or ADP alone, or in combination, did not have an effect on oligomerisation (Fig. 2 shows a representative experiment using BiP S^−^/K^−^).

### Highest ATPase activity is associated with S^+^ BiP variants

**Figure 3:**
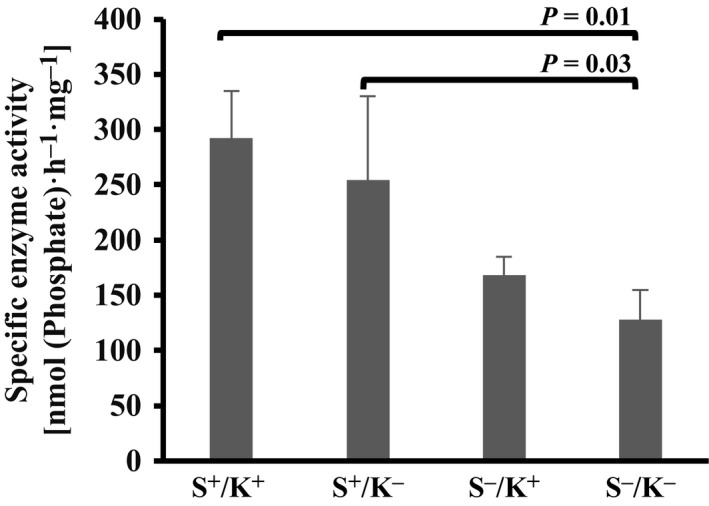
Biochemical characterisation of recombinant full‐length BiP and variant proteins thereof. ATPase activity of recombinant full‐length huBiP and three variant proteins was measured. ATPase activity assays were performed in a final volume of 160 μL containing 10 μg of protein with 30 mm HEPES‐KOH, pH 7.8, 150 mm NaCl, 20 μm ATP and 2 mm Mg^2+^. The proteins with the signal sequence had the highest activity compared to the other variants with no signal sequence. An unpaired Student's *t*‐test was performed, and significant differences were observed between full‐length huBiP and variant S^−^/K^−^ (*P = *0.01), as well as between variants S^−^/K^+^ and S^−^/K^−^ (*P = *0.03). Specific enzyme activity and respective standard deviation (SD) values were calculated using data from three individual batches for each recombinant protein variant.

The impact of the presence or absence of the signal sequence and/or KDEL on the ATPase activity of huBiP variants was tested using triplicate samples of independently purified protein batches. BiP that retained the signal sequences (BiP S^+^/K^+^ and S^+^/K^−^) had significantly higher ATPase activity than BiP S^−^/K^+^ and S^−^/K^−^ without the signal sequence (Fig. 3). BiP S^+^/K^−^ lacking the KDEL ER retention sequence also showed a trend towards a lower ATPase activity than full‐length (S^+^/K^+^) BiP, but the difference was not found to be significant (Fig. 3). These findings suggest that all recombinant proteins were produced and purified as functional proteins and that the signal sequence modulates ATPase activity.

### Low concentrations of divalent cations efficiently stimulate the ATPase activity of BiP

Initial experiments characterising the ATPase activity of huBiP variants were performed in the presence of millimolar concentrations of Mg^2+^, as previously published 7. While Mg^2+^ is considered the preferred divalent cation for stimulating BiP ATPase activity, the role of Mg^2+^ as co‐factor may be substituted by Mn^2+^. Therefore, we compared the effects of magnesium and manganese cations on the ATPase activity of S^+^/K^+^ and S^−^/K^−^ BiP over a wide range of concentrations (0–10 mm).

**Figure 4:**
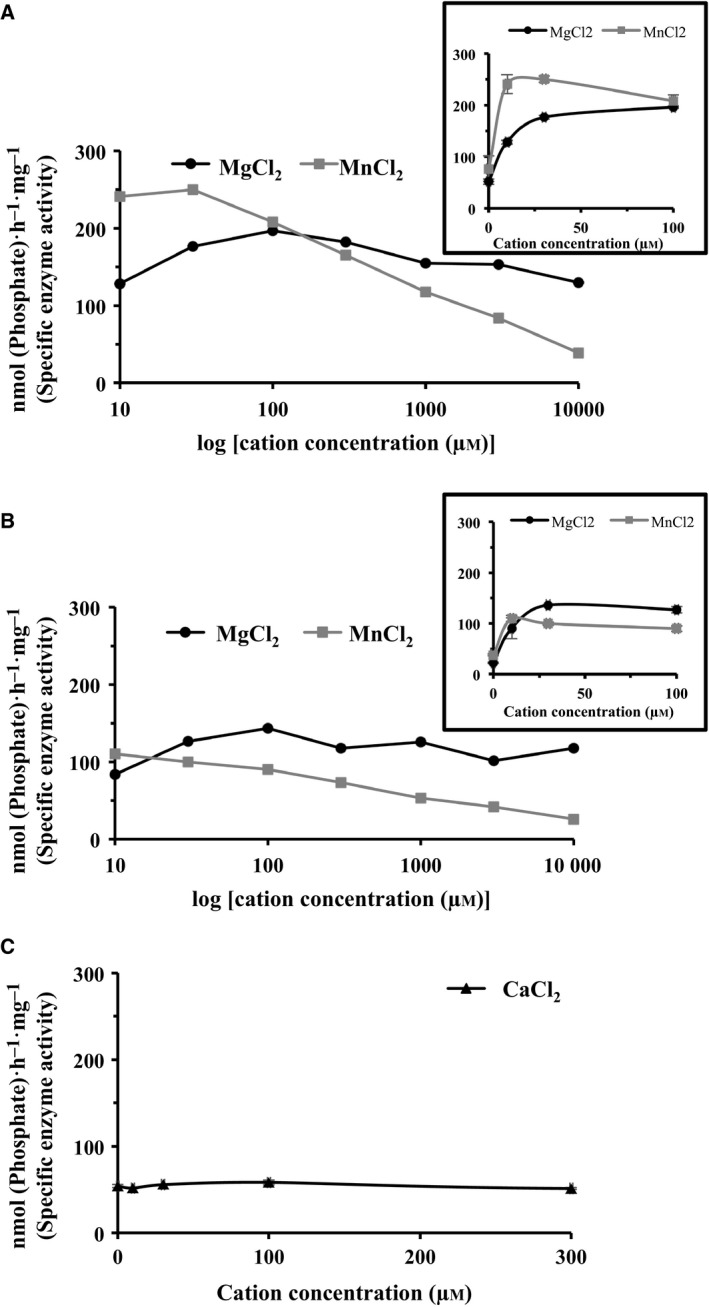
Effects of divalent cations on the ATPase activity of BiP S^+^/K^+^ and S^−^/K^−.^ To test the influence of divalent cations on the BiP ATPase activity, increasing amounts of MgCl_2_, MnCl_2_ and CaCl_2_ were added to the reaction mixture containing 20 μm ATP and 10 μg protein. ATPase assays were carried out in triplicate in a range from 0 to 10 mm of cations and representative results (mean of the values and SD) of such analyses are presented. MgCl_2_ and MnCl_2_ stimulated the ATPase activity of BiP with optimal concentrations of 100 and 25 μm, respectively. At higher concentrations of ≥ 1 mm, MgCl_2_ still stimulated ATPase activity but to a slightly lesser extent, whereas MnCl_2_ at concentration ≥ 1 mm failed to stimulate BiP ATPase activity and even inhibited it slightly. In contrast, calcium did not stimulate or inhibit the ATPase activity of BiP S^+^/K^+^. (A) BiP S^+^/K^+^ ATPase activity in the presence of Mg^2+^ and Mn^2+^ using a logarithmic scale for the cation presentation. The small inset shows ATPase activity of BiP S^+^/K^+^ at lower concentrations (0–100 μm) of Mg^2+^ and Mn^2+^ presenting the cation concentrations as a linear scale. (B) BiP S^−^/K^−^ ATPase activity in the presence of Mg^2+^ and Mn^2+^ with a logarithmic scale for the cations being used. The inset is the close‐up view of BiP S^−^/K^−^ ATPase activity at lower concentrations (0–100 μm) of Mg^2+^ and Mn^2+^ using a linear scale presentation of cation concentrations. (C) BiP S^+^/K^+^ ATPase activity in the presence of Ca^2+^ using a linear scale.

Concentrations of 10 and 30 μm Mg^2+^ efficiently stimulated the ATPase activity of S^+^/K^+^ and S^−^/K^−^ BiP (~ 175 nmol·h^−1^·mg^−1^ protein, Fig. 4A,B plus insets). Interestingly, at these low concentrations the stimulation of ATPase by Mn^2+^ was even more efficient than Mg^2+^, provoking a specific enzyme activity of up to 250 nmol·h^−1^·mg^−1^ of S^+^/K^+^ huBiP with Mn^2+^. However, at 100 μm or above, Mn^2+^ stimulates the ATPase activity of huBiP less efficiently than at 10–30 μm concentrations. It is important to note that concentrations of 20–50 μm of Mn^2+^ were found in normal brain, whereas concentrations of 60–150 μm of Mn^2+^ may already represent pathological levels 25. Increasing the Mn^2+^ concentration reduced the stimulatory effect of the divalent cation even further, such that at 10 mm, Mn^2+^ stimulation of BiP ATPase was no longer detected (Fig. 4A,B). In contrast, Mg^2+^ stimulated the ATPase in very wide range from 10 to 10 mm concentrations and did not have any inhibitory effect (Fig. 4A,B). Interestingly, the concentration of free Mg^2+^ was determined to be 0.8–1.2 mm in the ER 26. It is important to note that the huBiP ATPase did not show a clear maximal activity in this wide concentration range of Mg^2+^, and the difference between the ATPase activities at 100 μm, when compared to that measured at 10 mm, was only 15% (Fig. 4A,B). Both Mg^2+^ and Mn^2+^ cations stimulated the ATPase activity of BiP with optimal concentrations of approximately 50 and 25 μm, respectively. Calcium did not have any stimulatory effect on the ATPase activity of BiP S^+^/K^+^ (Fig. 4C).

### Preferential interaction of BiP variants with ADP rather than ATP

**Figure 5:**
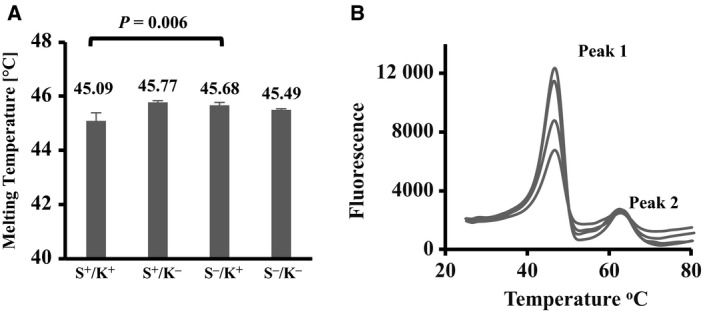
Biophysical characterisation of recombinant full‐length huBiP and variant proteins thereof. (A) Thermal stability analyses of recombinant full‐length huBiP and three variant proteins thereof were performed using DSF. The reaction mixtures contained 1 μm protein in 30 mm HEPES‐KOH, pH 7.8, 150 mm NaCl and 1× Sypro orange. The melting temperatures of the full length and variant proteins are in the range of 45.1–45.8 °C. The calculated *T*_m_ (e.g. the maximum of the first derivative of the raw data) is shown as a mean of triplicate experiments of three individual batches for each protein variants with respective SD error bars. An unpaired Student's *t*‐test was performed, and a significant difference was observed between S^+^/K^+^ and S^−^/K^−^ (*P = *0.006). (B) Thermal denaturation profile of recombinant full‐length huBiP and variants with omitted N‐ or C‐terminal amino acids. The first peak (46.6 °C for S^+^/K^+^, 46.8 °C for S^+^/K^−^, 46.8 °C for S^−^/K^+^ and 46.6 °C for S^−^/K^−^) indicates the unfolding of the N terminus of these proteins, whereas the second peak (62.75 °C for S^+^/K^+^, 62.2 °C for S^+^/K^−^, 63 °C for S^−^/K^+^ and 62.4 °C for S^−^/K^−^) represents the unfolding of the C‐terminal domain of these proteins 8. The thermal denaturation profiles of full‐length huBiP and variant proteins suggest that the N‐ and C termini of these proteins are properly folded. Representative melting curves with calculated *T*_m_ are shown as a mean of three individual batches for each protein variant. Their low values of SDs of the melting temperatures suggest a very good reproducibility of the experiments (data not shown).

Physical interactions of proteins with their ligands, cofactors and substrates can stabilise interacting protein domains and thus cause an increase in the melting temperature of proteins. In keeping with the methods of others 27, DSF was used to analyse the melting temperature of all BiP variants in the presence or absence of nucleotides. In the absence of ADP or ATP, the melting temperatures of the BiP variants tested here ranged from 45.1 to 45.8 °C (Fig. 5A). Thermal denaturation profiles demonstrated the unfolding of the N‐terminal (peak 1) and C‐terminal (peak 2) of these proteins (Fig. 5B) similar to previously published results 8. The N‐terminal denaturing temperatures were as follows: S^+^/K^+^: 46.6 °C, S^+^/K^−^: 46.8 °C, S^−^/K^+^: 46.8 °C and S^−^/K^−^: 46.6 °C, while C‐terminal denaturing temperatures were as follows: S^+^/K^+^: 62.8 °C, S^+^/K^−^: 62.2 °C, S^−^/K^+^: 63 °C and S^−^/K^−^: 62.4 °C.

**Figure 6:**
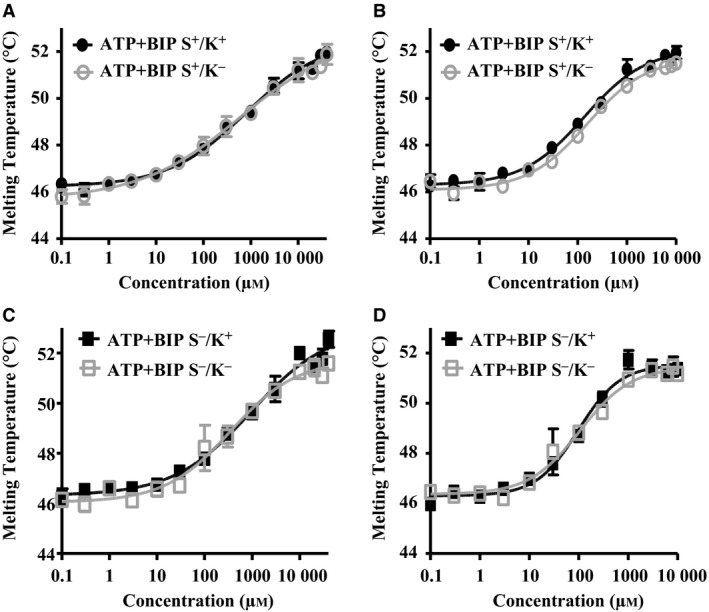
Nucleotide binding of huBiP variants. In DSF experiments presented here, 1 μm protein was incubated in 30 mm HEPES‐KOH, pH 7.8, 150 mm NaCl and 1× Sypro orange. The melting curve of the samples was measured from 25 to 95 °C with 1 °C increments increase in temperature. (A) The interaction of BiP S^+^/K^+^ and BiP S^+^/K^−^ with ATP was measured in the absence of divalent cations. The denaturation profiles of the proteins were studied in the presence of increasing amounts of ATP (0–40 mm). (B) The interaction of BiP S^+^/K^+^ and BiP S^+^/K^−^ with ADP was determined without divalent cations. Denaturation profiles of the proteins were studied in the presence of increasing amounts of ADP (0–10 mm). (C) The interaction of BiP S^−^/K^−^ and BiP S^−^/K^+^ with ATP was measured with no divalent cations present. The denaturation profiles of the proteins were studied in the presence of increasing amounts of ATP (0–40 mm). (D) The interaction of huBiP BiP S^−^/K^−^ and huBiP S^−^/K^+^ with ADP was determined without divalent cations. Denaturation profiles of the proteins were studied in the presence of increasing amounts of ADP (0–10 mm). In each diagram, the melting temperatures of the proteins as indicated (*y*‐axis linear scale) dependent on the nucleotide concentrations (*x*‐axis in logarithmic scale) are presented. The *K*_d_ values were calculated using graphpad prism software under the assumption of a simple cooperative model.

Differential scanning fluorimetry was then used to study the binding affinity of ADP or ATP for BiP variants, in the absence of cations (Fig. 6, summarised in Table 1). The *K*_d_ of ATP of all variants was as follows: S^+^/K^+^, 766 μm; S^−^/K^−^, 352 μm; S^+^/K^−^, 610 μm; S^−^/K^+^, 945 μm (Fig. 6A to 6D, Table 1). ADP values were lower, that is S^+^/K^+^, 138 μm; S^−^/K^−^, 107 μm; S^+^/K^−^, 188 μm; S^−^/K^+^, 98 μm. These findings indicate that in the absence of divalent cations, BiP variants have a higher binding affinity for ADP than for ATP.

### Divalent cations modulate nucleotide binding properties of BiP

**Figure 7:**
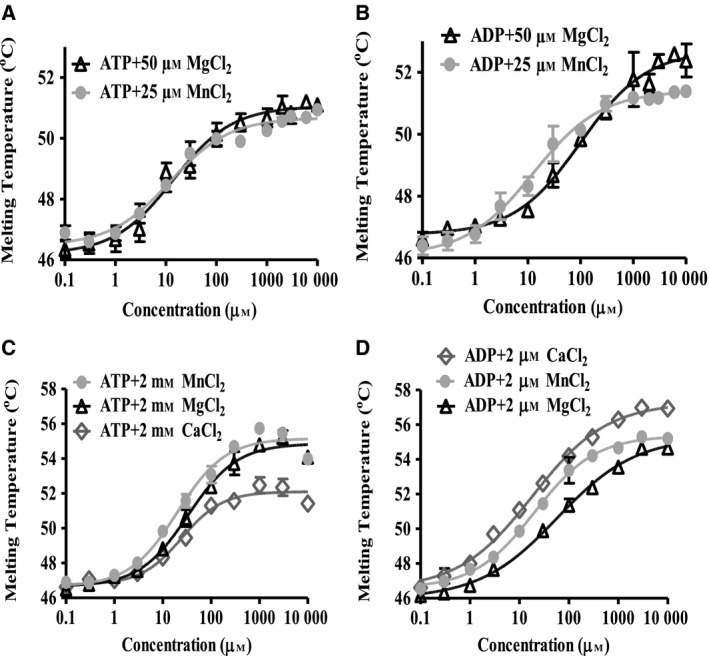
Effect of divalent cations on BiP S^+^/K^+^ and BiP S^−^/K^−^ nucleotide binding. In these DSF experiments, 1 μm protein was incubated in 30 mm HEPES‐KOH, pH 7.8, 150 mm NaCl and 1× Sypro orange. The melting curve of the samples was measured from 25 to 95 °C with 1 °C increments increase in temperature. (A) The interaction of BiP S^+^/K^+^ in the presence of increasing amounts of ATP (0–10 mm) was studied using 50 μm Mg^2+^ and 25 μm Mn^2^ as indicated. (B) Interaction of BiP S^+^/K^+^ with increasing concentrations of ADP (0–10 mm) in the presence 50 μm Mg^2+^ and 25 μm Mn^2+^ was measured. (C) The binding of BiP S^+^/K^+^ to increasing amounts of ATP (0–10 mm) having 2 mm of divalent cations (Mg^2+^, Mn^2+^ and Ca^2 +^ as indicated) present was measured. (D) The interaction of BiP S^+^/K^+^ with increasing amounts of ADP (0–10 mm) in the presence of 2 mm of divalent cations (Mg^2+^, Mn^2+^ and Ca^2 +^) was analysed. In each diagram, the melting temperatures of the proteins as indicated (*y*‐axis linear scale) dependent on the nucleotide concentrations (*x*‐axis in logarithmic scale) are presented. The *K*_d_ values were calculated using graphpad prism software under the assumption of a simple cooperative model.

The optimisation of the ATPase assay conditions showed a strict dependence on the presence of Mg^2+^ and Mn^2+^ divalent cations, whereas Ca^2+^ did not support ATPase activity (Fig. 4A–C). To determine the effect of divalent cations on nucleotide binding in more detail, we focused on the longest (S^+^/K^+^) and shortest (S^−^/K^−^) variants, as these represent the variants with the most significant differences in their enzyme activities. In the presence of divalent cations (Mg^2+^, Mn^2+^, Ca^2+^), there was a several‐fold increase in the affinities of ATP and ADP with both variants, at low (50 μm of Mg^2+^ and 25 of μm Mn^2+^, Fig. 7A,B, summarised in Table 1), and high divalent cation concentrations (2 mm each of Mg^2+^, Mn^2+^ and Ca^2+^, Fig. 7C,D, also see Table 1). Thus, the affinity of the two BiP variants for both nucleotides increased 2‐ to 10‐fold in the presence of divalent cations (Table 1).

In the presence of 2 mm of Mg^2+^, Mn^2+^ or Ca^2+^, full‐length BiP bound to ATP with *K*_d_ values of 44, 27 or 30 μm, respectively, whereas BiP S^−^/K^−^ had at same divalent cation concentrations, *K*_d_ values of 13, 5 and 10 μm, respectively, suggesting that under these conditions, S^−^/K^−^ BiP has a greater affinity than S^+^/K^+^ BiP for ATP. The interaction of the two BiP variants with ADP was similarly influenced by divalent cations. S^+^/K^+^ BiP binds to ADP at concentrations of 2 mm of Mg^2+^, Mn^2+^ or Ca^2+^ with *K*_d_ values of 59, 21 and 19 μm, respectively, whereas ADP interacts with BiP S^−^/K^−^ in the presence of the same divalent cations with *K*_d_ values of 27, 15 and 5 μm, respectively. Interestingly, these data revealed that under the same cation conditions, full‐length BiP had lower affinity for ATP and ADP than S^−^/K^−^ BiP.

In summary, these studies demonstrate that for both BiP variants tested, the ratio of *K*_d_ (ATP)/*K*_d_ (ADP) is larger than 1 in the presence of Ca^2+^ (ratio: 1.6–2), whereas the ratio of *K*_d_ (ATP)/*K*_d_ (ADP) is smaller than 1 with Mg^2+^ (ratio: 0.23–0.37). These findings suggest that in presence of Ca^2+^, BiP binds with higher affinity to ADP than to ATP, while in the presence of Mg^2+^, BiP shows stronger binding to ATP than to ADP. This could explain at least in part why Ca^2+^ does not support BiP's ATPase activity since in the presence of Ca^2+^ it is difficult for ATP to replace enzyme‐bound ADP after hydrolysis, to allow its reloading in the following ATPase reaction. In contrast, the higher affinity of BiP for ATP than for ADP in the presence of Mg^2+^ would suggest that with this divalent cation, ATP can easily replace enzyme‐bound ADP. The situation for Mn^2+^ is less clear, and the ratio is about one for full‐length BiP and BiP S^−^/K^−^ (ratio: 1.3 and 0.86), suggesting that ATP might be able to replace enzyme‐bound ADP in the presence of Mn^2+^ and that it would happen more easily than in the presence of Ca^2+^. From these nucleotide binding data, a ranking of the divalent cations for stimulating BiP enzyme activity would be Mg^2+^ > Mn^2+^ > Ca^2+^, with the latter being most likely unable to support BiP ATPase activity.

### Kinetic analysis of ATPase activity of BiP

**Figure 8:**
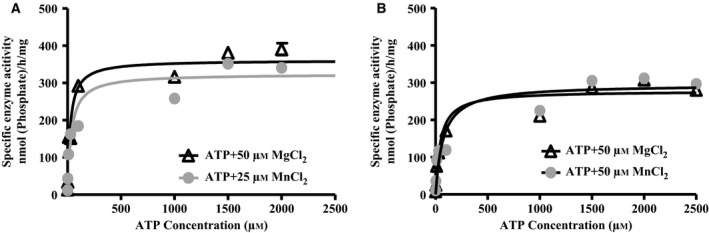
BiP ATPase activities in the presence of low concentrations of divalent cations. ATPase activity of huBiP was measured in the presence of MgCl_2_ (50 μm) and MnCl_2_ (25 μm). Samples were prepared with a reaction volume of 160 μL containing 10 μg of protein, 30 mm HEPES‐KOH, pH 7.8, 150 mm NaCl, either 50 μm MgCl_2_ or 25 μm MnCl_2_ and increasing concentrations of ATP (0–3 mm). Experiments were repeated twice in each case but only one representative set of experiments is presented. (A) BiP S^+^/K^+^ ATPase activity in the presence of 50 μm Mg^2+^ and 25 μm Mn^2+^. (B) BiP S^−^/K^−^ ATPase activity in the presence of 50 μm of Mg^2+^ and 25 μm of Mn^2+^. The *K*_m_ and *V*_max_ values were calculated using the Michaelis‐Menten equation and graphpad prism software.

We next addressed the question of whether or not the kinetics of ATPase activity was significantly different, when S^+^/K^+^ and S^−^/K^−^ BiP variants were compared. We were particularly interested in ATPase activities occurring at submillimolar concentrations of Mn^2+^, as these represent normal physiological levels found within the human brain. Specifically, a concentration of 20.0–52.8 μm is recommended for the *in vitro* modelling of normal brain Mn^2+^, while concentrations of 60.1–158.4 μm of Mn^2+^ can be considered to represent threshold pathological levels 25. Based on these findings and on our Mg^2+^‐ and Mn^2+^‐dependent ATPase activity data (Fig. 4), we used 50 μm of Mg^2+^ and 25 μm of Mn^2+^ for kinetic profiling. In the presence of either cation, the ATPase reaction showed a hyperbolic function for both BiP variants (Fig. 8). The *V*_max_ values of both BiP variants with both divalent cations were similar (Table 2). However, in the presence of 50 μm Mg^2+^, S^+^/K^+^ BiP had approximately a 20% higher *V*_max_ than S^−^/K^−^ BiP under these conditions, whereas the three other *V*_max_ values were very consistent and they varied less than 5% from each other (Table 2).

**Table 2:** Kinetic Analysis of BiP ATPase activity

Protein	Divalent cation	*K*_M_ [μm]	Standard deviation(±)	*V*_max_ [nmol·h^−1^·mg^−1^]	Standard deviation(±)
S^+^/K^+^	MgCl_2_ 50 μm	18	9.9	371	14
	MnCl_2_ 25 μm	38	4.2	311	18
S^−^/K^−^	MgCl_2_ 50 μm	61	21	293	21
	MnCl_2_ 25 μm	66	9.9	307	18

In contrast, the *K*_M_ values differed by a factor of up to 3.7 (Fig. 8 and Table 2). S^+^/K^+^ BiP had a *K*_M_ value of 18 μm in the presence of 50 μm of Mg^2+^, which was the lowest of all *K*_M_ values measured. Interestingly, this *K*_M_ is very similar to the *K*_d_ value of 14 μm for ATP, suggesting that in the presence of 50 μm of Mg^2+^, the binding of ATP to full‐length BiP is the major contributing factor for its ATPase activity (compare respective data in Tables 1 and 2). In the presence of 25 μm Mn^2+^, the *K*_M_ of 38 μm of full‐length BiP is twice as high as the *K*_M_ value with 50 μm of Mg^2+^. Interestingly, the *K*_M_ of full‐length BiP is also three times higher than the *K*_d_ of 12 μm for ATP under the same Mn^2+^ conditions (compare respective data in Tables 1 and 2), revealing the possibility of another mechanism being the rate‐limiting step in the ATPase reaction under this condition, for example, the formation of the transition complex or the possibility that a secondary nucleotide binding site may modulate the ATPase reaction.

For the S^−^/K^−^ variant, at 50 μm of Mg^2+^ and 25 μm Mn^2+^, the *K*_M_ values of 61 and 66 μm, respectively, are very similar, but both are higher (about 2–3 times) than the values found for full‐length BiP, suggesting that the shorter BiP variant needs higher ATP concentrations than full‐length BiP to reach *V*_max_. Interestingly, the difference between the measured *K*_M_ values for BiP S^−^/K^−^ and the apparent *K*_d_ values for ATP is 6‐ and 33‐fold higher in the presence of 50 μm Mg^2+^ or 25 μm Mn^2+^, respectively. This suggests that in contrast to full‐length BiP in the presence of Mg^2+^, additional steps, especially in the case of BiP S^−^/K^−^ with 25 μm Mn^2+^, are controlling the ATPase activity of the shortest variant. On the other hand, in the presence of 25 μm Mn^2+^, the determined *V*_max_ (307 nmol·h^−1^·mg^−1^) of BiP S^−^/K^−^ is comparable to the *V*_max_ (293 nmol·h^−1^·mg^−1^) of full‐length BiP, indicating that at high ATP concentrations, ATP hydrolysis is no longer influenced by this apparent inhibitory mechanism, which usually would regarded as a competitive inhibition.

## Discussion

B‐cell immunoglobulin binding protein is an ER‐resident chaperone with several intra‐ and extracellular functions. The intracellular functions of BiP include calcium binding, folding of unfolded protein, supporting transport of proteins into the ER, trafficking of misfolded proteins for degradation and regulating the unfolded protein response, whereas in its extracellular activities, it interacts with the immune cells and has anti‐inflammatory properties in the context of RA 1, 28, 29. All or most of these functions require BiP‐ATP or BiP‐ADP interactions 1, 29. Nucleotide binding of BiP causes a conformational change of the protein and assists in its binding to newly synthesised peptides to catalyse their proper folding. To study the basic functions of huBiP, we expressed four variants, including the full‐length protein and those lacking the signal peptide, the KDEL ER retention sequence or both. The purified recombinant proteins show the correct size, varying slightly, depending on the presence or absence of signal/KDEL peptides. Here, it is important to note that the presence of six histidines has no effect on the biochemical properties of BiP, as previously reported 18.

Native BiP exits as a monomer, dimer and in higher oligomeric forms 17. All these forms were also determined for the full‐length recombinant BiP, and its variants studied here as shown by the native PAGE, which, with the DSF data, suggests that these recombinant proteins are properly folded. These higher oligomeric forms may act as a reservoir and can be instantly converted into active monomeric forms upon demand 30. Binding of ATP can also convert BiP dimers to monomers 31, 32. Full‐length huBiP and the shortest version were tested using native gels for their oligomerisation in response to nucleotides and divalent cations and were shown to behave similarly in the presence of ATP alone or in the presence of ATP and divalent cations. This effect was not seen with ADP. Previous reports showed that ATPase activity and nucleotide binding properties are conserved functions of heat shock proteins 33 and that divalent cations such as Mg^2+^, Mn^2+^ and Ca^2+^ impact on the ATPase activity of non huBiP 1, 7, 18. To further characterise the purified recombinant huBiP variants, we investigated their melting temperatures including the melting temperature shift in the presence of nucleotides using DSF and their ATPase activities. We determined that all variants had a similar melting temperature and that the melting curves of all four variants were similar to those previously described for nonhuman S^−^/K^+^ BiP 8. For the latter, it has been reported that the thermal denaturing temperatures or melting temperatures of the N‐terminal and the C‐terminal were 46.2 and 67 °C, respectively. Both melting temperatures are comparable to those of the different huBiP variants reported here. It is important to note that unfolding of the N‐terminal ATP‐binding domain happens first followed by the unfolding of the C‐terminal SBDs as previously described 8.

B‐cell immunoglobulin binding protein has a low Mg^2+^‐dependent ATPase activity 7, 34. Various reports have described the influence of specific divalent cations on the functions of BiP and especially their impact on its ATPase activity 7, 18. Therefore, we decided to study the effect of cations on the ATPase activity of huBiP variants, focusing on full‐length (S^+^/K^+^) BiP and the S^−^/K^−^ variant. Interestingly, in our assays, both magnesium and manganese ions very efficiently and to a comparable extent stimulated the ATPase activity of full‐length BiP and the S^−^/K^−^ variant, at micromolar concentrations. In contrast, Ca^2+^ did not support the ATPase activity of full‐length huBiP, which is in agreement with the results previously described for mammalian BiP 8, 18. This lack of stimulation in the presence of calcium could be due to the inhibition of nucleotide exchange, for example, a higher affinity of BiP to ADP than to ATP in the presence of Ca^2+^, which is consistent with our data and those previously described 7, 8, 18. The efficiency of Mg^2+^ and Mn^2+^ to stimulate huBiP ATPase at low micromolar divalent ion concentrations was surprising, since previously the optimal Mg^2+^ concentration for mammalian BiP was described as 1 mm and it was reported that Mn^2+^ is significantly less efficient than Mg^2+^ at stimulating ATPase activity 7, 18. Perhaps previous studies missed the stimulation if submillimolar concentrations of cations were not examined closely 7, 18. However, it is important to note that the range of efficient stimulation of huBiP ATPase activity by Mn^2+^ mirrors the physiological concentrations found in human brain. The concentration of Mg^2+^ in the ER has been described to be higher than that of Mn^2+^ and is in the range of 10 mm, but only a fraction of this Mg^2+^ is available as free cation 35. Although the concentration of free Mg^2+^ in the ER has not yet been determined, it is thought to be similar as that in the cytoplasm and other cellular compartments where submillimolar concentrations of free Mg^2+^ were measured 35. It is important to note that the potential for either cation to influence BiP ATPase activity *in vivo* must depend on the particular tissue and cellular context 25.

Consistent with previous reports, DSF showed a two‐phase melting‐point profile of huBiP with the early melting point most likely being due to the melting of the N‐terminal NBD of hamster BiP 8, which is consistent with the finding that divalent cations as well as ATP and ADP binding stabilise this domain. Therefore, we also used DSF to study BiP‐ligand interactions and to measure the dissociation constants (*K*_d_) of all the variants of BiP for ATP and ADP including the effect of cations (Mg^2+^, Mn^2+^ and Ca^2+^) on the nucleotide binding, as previously described 27. In the absence of divalent cations, the *K*_d_ values of all the variants for ADP are lower than *K*_d_ for ATP, indicating that ADP has a higher binding affinity than ATP under these conditions. The addition of divalent cations Mg^2+^, Mn^2+^ and Ca^2+^ reduced the *K*_d_ values of huBiP for both ATP and ADP by up to 80%, confirming that cations act as cofactors in binding of BiP variants to these nucleotides 8. However, the K_d_ values of BiP for ATP and ADP appear to vary significantly, depending on the source of the protein and the methods used to determine the binding of BiP and other heat shock proteins to these nucleotides 36. Heat shock cognate protein 71 kDa prepared from bovine brain or expressed in bacteria was reported to have *K*_d_ values for ADP or ATP ranging from 10^−5^ to 10^−8^ 36. The values for huBiP variants, determined here, fall within that range. Importantly, we report here for the first time, the influence of the concentration of divalent cations on the binding affinity of the BiP nucleotide interactions.

**Figure 9:**
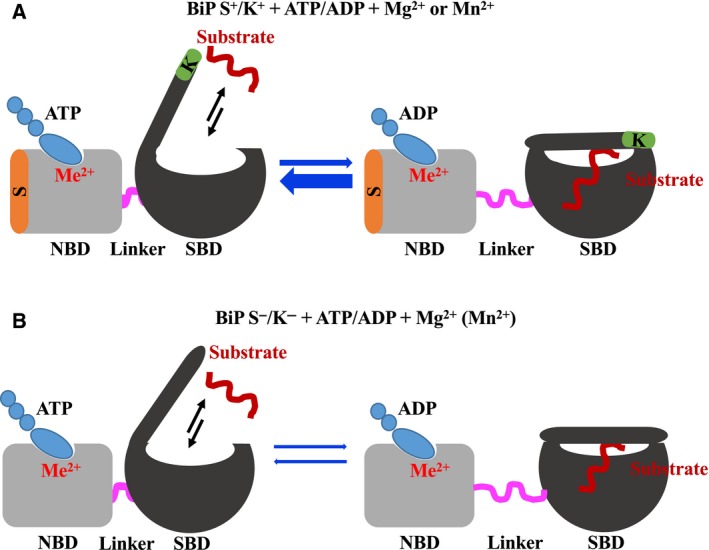
Model illustrating BiP‐ATP‐ and BiP‐ADP‐bound conformation. (A) Full‐length BiP tends to be in open conformation in the ATP‐bound state (indicated by thick blue arrow) in the presence of metal ions (Me^2+^), enabling easy exchange of ATP. (B) BiP S^−^/K^−^ tends to be in a closed conformation in the ADP‐bound state in the presence of Ca^2+^ and the binding of ADP is tighter.

A detailed analysis showed that at low concentrations of divalent cation (50 μm Mg^2+^), the *K*_d_ value of S^+^/K^+^ and S^−^/K^−^ for ADP is at least seven times higher than the *K*_d_ of S^+^/K^+^ and S^−^/K^−^ for ATP, meaning that the affinity of these proteins for ATP is much greater than for ADP and that in the presence of Mg^2+^, the exchange of ADP happens easily. In contrast, the presence of manganese even at a low concentration results in ADP being tightly bound to huBiP, hindering its ATPase activity at least to some extent. However, as previously reported for higher cation concentrations, the affinity of BiP for ATP is still higher than it is for ADP, such that an exchange of ADP to ATP should still happen. Thus, the lower efficiency of huBiP activity at low ATP concentrations in the presence of Mn^2+^ (meaning higher *K*_M_) can be explained by the affinities of huBiP for ATP or ADP, at least in part. However, the higher *K*_M_ of S^−^/K^−^ cannot be explained simply by the binding model. Losing these two peptides may result in a conformational hindrance or a kinetic problem affecting the exchange between the two stages, that is the ATP‐ and the ADP‐bound forms, or it may cause a conformational change that affects the hydrolysis of ATP, since the binding of ATP is not the limiting factor for the reaction of S^−^/K^−^ variant of huBiP (see model in Fig. 9). The stimulation of the ATPase activity could be caused by the signal sequence acting as an intramolecular D‐type domain, or it may even act as a substrate and that this activity is enhanced by the C‐terminal KDEL sequence. It is worth remembering that classically, BiP binds to polypeptides and is involved in their translocation into the ER. BiP interacts with the J‐domain of the Sec63p, a component of the Sec complex that plays a vital role in the translocation of polypeptides across the ER membrane 37. BiP in the ADP‐bound confirmation blocks the translocon and prevents protein translocation and Ca^2+^ leakage, whereas the ATP‐bound state opens the translocon and assists in translocation 38. BiP has more preference towards aliphatic amino acids alanine, valine, leucine and proline. Leucine is the most preferred amino acid for binding to BiP 39. Due to the presence of six leucines in the signal sequence of BiP and the hydrophobic nature of the signal sequence itself, there exists a possibility of intermolecular or intramolecular binding via the signal sequence, perhaps leading to an enhanced ATPase activity of huBiP variants that retain the signal sequence.

Furthermore, when considering the enzyme reaction, the affinities of each flavour of huBiP for ATP and ADP are very important. The ATP‐bound state has a weak affinity for substrates (peptides) and the ADP‐bound state has a higher affinity for substrates, locking the peptides in the SBD for folding (see model proposed in Fig. 9). In the presence of Mg^2+^, S^+^/K^+^ and S^−^/K^−^ both prefer to be in the ATP‐bound conformation, while under Ca^2+^ conditions, S^−^/K^−^ and S^+^/K^+^ both favour the ADP‐bound state due to their higher affinity for ADP than for ATP. For Mn^2+^, the situation is less obvious, but it may depend on its concentration, that is low (micromolar) and physiological, or high (millimolar) possibly pathological.

At concentrations 50 μm of Mg^2+^, the *K*_d_ value of S^+^/K^+^ for ATP is similar to the *K*_M_ value obtained suggesting that ATP and ADP can freely exchange at the BiP nucleotide binding site. The binding of ATP to S^+^/K^+^ does not hinder the enzyme activity. In contrast, at low concentration, 25 μm, of Mn^2+^, the *K*_d_ value of S^+^/K^+^ for ATP is lower compared to the *K*_m_ value obtained, revealing that ATP is tightly bound but that the hydrolysis of ATP to ADP might be slower. The increased *K*_M_ value suggests that the enzyme needs higher concentration of nucleotide (ATP) to reach the same *V*_max_. At low divalent cation concentration, 50 μm of Mg^2+^ and 25 μm of Mn^2+^, the *K*_d_ values of S^−^/K^−^ for ATP are lower compared to the *K*_M_ values obtained suggesting that ATP is tightly bound but that the hydrolysis of ATP to ADP is slow. This is also consistent with ATPase assay data suggesting these variants without the signal sequence and KDEL are less active; that is, they need more nucleotide (ATP) to reach the same *V*_max_. The enzyme activity might be hindered by the tight binding of ATP to S^−^/K^−^ or kinetic hinderance of the formation of the ATP‐containing transition complex or its changes towards an ADP‐bound complex including the hydrolysis of ATP and the formation of ADP (see Fig. 9).

The ATPase activity depends on the pH and the charge of the NBD of BiP. To study the kinetic parameters of huBiP, ATPase enzyme assays in the presence of increasing ATP concentration were performed and *K*_M_ and *V*_max_ values were determined. Interestingly, the specific enzyme activity and *V*_max_ values of ~ 300 nmol·h^−1^·mg^−1^ for huBiP S^−^/K^−^ in comparison with the literature are very similar although different set‐ups and choices of buffers were used. Wei and Hendershot measured the ATPase activity of recombinant hamster BiP purified from bacteria and reported a *V*_max_ of 5.2 pmol of ATP hydrolysed·min^−1^·μg^−1^ (312 nmol·h^−1^·mg^−1^), whereas 4.7 pmol of ATP hydrolysed·min^−1^·μg^−1^ (282 nmol·h^−1^·mg^−1^) was described for BiP purified from canine pancreas 7, which are comparable to each other. In contrast, the *K*_M_ values for these purified enzymes seem to vary more, since published values of *K*_M_ = 1.48 ± 0.1 μm (purified recombinant haBiP 18, *K*_M_ = 0.1 μm (BiP purified from canine pancreas 7) and *K*_M_ of 0.4 μm for ATP (recombinant murine BiP 40 have been previously reported, whereas we found *K*_M_ of 18–66 μm depending on the Mg^2+^ and Mn^2+^ concentrations. The difference in these value could depend on the assays such as radioactive and nonradioactive assay system as well as the buffer conditions used but also that here, low μm concentrations of divalent cations and not mm concentrations of Mg^2+^ were present in the assays.

## Conclusion

We have purified recombinant huBiP variants from *E. coli,* and these variants behave similarly to previously analysed BiP in terms of size, melting temperatures and conformation. Consistent with previous reports, divalent cations influence the binding of nucleotides and ATPase activity of purified huBiP variants and they showed similar *V*_max_ values to previously published studies. Interestingly, we found very efficient functional interactions of BiP with Mn^2+^ at low physiological concentration. However, these differed in some enzyme parameters such as K_d_ for nucleotides and *K*_M_ values. All variants bind ADP more tightly than ATP. Interestingly, the presence of signal sequence significantly influences the enzyme characteristics of huBiP.

## Conflict of interest

The authors declare no conflict of interest.

## Author contributions

SB performed the research. SD guided and trained SB during her experiments. UF and HPN designed and guided the research project. SB, SD, UF and HPN wrote the manuscript.

## References

[1] Wang J , Lee J , Liem D and Ping P (2017) HSPA5 Gene encoding Hsp70 chaperone BiP in the endoplasmic reticulum. Gene 618, 14–23. 28286085 10.1016/j.gene.2017.03.005 PMC5632570

[2] Lee AS (2007) GRP78 Induction in Cancer: therapeutic and Prognostic Implications. Cancer Res 67, 3496–3499. 17440054 10.1158/0008-5472.CAN-07-0325

[3] Pidoux AL and Armstrong J (1993) The BiP protein and the endoplasmic reticulum of *Schizosaccharomyces pombe*: fate of the nuclear envelope during cell division. J Cell Sci 105, 1115–1120. 8227200 10.1242/jcs.105.4.1115

[4] Awad W , Estrada I , Shen Y and Hendershot LM (2008) BiP mutants that are unable to interact with endoplasmic reticulum DnaJ proteins provide insights into interdomain interactions in BiP. Proc Natl Acad Sci 105, 1164–1169. 18203820 10.1073/pnas.0702132105 PMC2234109

[5] Mayer MP and Bukau B (2005) Hsp70 chaperones: cellular functions and molecular mechanism. Cell Mol Life Sci 62, 670–684. 15770419 10.1007/s00018-004-4464-6 PMC2773841

[6] Bertelsen EB , Chang L , Gestwicki JE and Zuiderweg ERP (2009) Solution conformation of wild‐type *E. coli* Hsp70 (DnaK) chaperone complexed with ADP and substrate. Proc Natl Acad Sci 106, 8471–8476. 19439666 10.1073/pnas.0903503106 PMC2689011

[7] Kassenbrock CK and Kelly RB (1989) Interaction of heavy chain binding protein (BiP/GRP78) with adenine nucleotides. EMBO J 8, 1461–1467. 2670554 10.1002/j.1460-2075.1989.tb03529.x PMC400975

[8] Lamb HK , Mee C , Xu W , Liu L , Blond S , Cooper A , Charles IG and Hawkins AR (2006) The affinity of a major Ca^2+^ binding site on GRP78 is differentially enhanced by ADP and ATP. J Biol Chem 281, 8796–8805. 16418174 10.1074/jbc.M503964200

[9] Ni M , Zhang Y and Lee AS (2011) Beyond the endoplasmic reticulum: atypical GRP78 in cell viability, signalling and therapeutic targeting. Biochem J 434, 181–188. 21309747 10.1042/BJ20101569 PMC3353658

[10] Marín‐Briggiler CI , González‐Echeverría MF , Munuce MJ , Ghersevich S , Caille AM , Hellman U , Corrigall VM and Vazquez‐Levin MH (2010) Glucose‐regulated protein 78 (Grp78/BiP) is secreted by human oviduct epithelial cells and the recombinant protein modulates sperm–zona pellucida binding. Fertil Steril 93, 1574–1584. 19296942 10.1016/j.fertnstert.2008.12.132

[11] Giusti L , Baldini C , Ciregia F , Giannaccini G , Giacomelli C , Feo FD , Sedie AD , Riente L , Lucacchini A , Bazzichi L *et al* (2010) Is GRP78/BiP a potential salivary biomarker in patients with rheumatoid arthritis? Proteomics Clin Appl 4, 315–324. 21137052 10.1002/prca.200900082

[12] Tsunemi S , Nakanishi T , Fujita Y , Bouras G , Miyamoto Y , Miyamoto A , Nomura E , Takubo T and Tanigawa N (2010) Proteomics‐based identification of a tumor‐associated antigen and its corresponding autoantibody in gastric cancer. Oncol Rep 23, 949–956. 20204278 10.3892/or_00000719

[13] Shields AM , Panayi GS and Corrigall VM (2012) A new‐age for biologic therapies: long‐term drug‐free therapy with BiP? Front Immunol 3, 1–8. 22566902 10.3389/fimmu.2012.00017 PMC3342250

[14] Brownlie RJ , Myers LK , Wooley PH , Corrigall VM , Bodman‐Smith MD , Panayi GS and Thompson SJ (2006) Treatment of murine collagen‐induced arthritis by the stress protein BiP via interleukin‐4‐producing regulatory T cells: a novel function for an ancient protein. Arthritis Rheum 54, 854–863. 16508967 10.1002/art.21654

[15] Kirkham B , Chaabo K , Hall C , Garrood T , Mant T , Allen E , Vincent A , Vasconcelos JC , Prevost AT , Panayi GS *et al* (2016) Safety and patient response as indicated by biomarker changes to binding immunoglobulin protein in the phase I/IIA RAGULA clinical trial in rheumatoid arthritis. Rheumatol 55, 1993–2000. 10.1093/rheumatology/kew287 PMC5854092 27498355

[16] Sou SN , Ilieva KM and Polizzi KM (2012) Binding of human BiP to the ER stress transducers IRE1 and PERK requires ATP. Biochem Biophys Res Commun 420, 473–478. 22446326 10.1016/j.bbrc.2012.03.030

[17] Laemmli UK (1970) Cleavage of structural proteins during the assembly of the head of the bacteriophage T4. Nature 227, 680–685. 5432063 10.1038/227680a0

[18] Wei J and Hendershot LM (1995) Cell biology and metabolism: characterization of the nucleotide binding properties and ATPase activity of recombinant hamster BiP purified from bacteria. J Biol Chem 270, 26670–26676. 7592893 10.1074/jbc.270.44.26670

[19] Čiplys E , Aučynaite A and Slibinskas R (2014) Generation of human ER chaperone BiP in yeast *Saccharomyces cerevisiae* . Microb Cell Fact 13. 10.1186/1475-2859-13-22 PMC3926315 24512104

[20] Ericsson UB , Hallberg BM , Detitta GT , Dekker N and Nordlund P (2006) Thermofluor‐based high‐throughput stability optimization of proteins for structural studies. Anal Biochem 357, 289–298. 16962548 10.1016/j.ab.2006.07.027

[21] Vivoli M , Novak HR , Littlechild JA and Harmer NJ (2014) Determination of protein‐ligand interactions using differential scanning fluorimetry. J Vis Exp 91, e51809. 10.3791/51809 PMC4692391 25285605

[22] Corrigall VM , Bodman‐Smith MD , Brunst M , Cornell H and Panayi GS (2004) Inhibition of antigen‐presenting cell function and stimulation of human peripheral blood mononuclear cells to express an antiinflammatory cytokine profile by the stress protein BiP: relevance to the treatment of inflammatory arthritis. Arthritis Rheum 4, 1164–1171. 10.1002/art.20134 15077298

[23] Zhang Y , Liu R , Ni M , Gill P and Lee AS (2010) Cell surface relocalization of the endoplasmic reticulum chaperone and unfolded protein response regulator GRP78/BiP. J Biol Chem 20, 15065–15075. 10.1074/jbc.M109.087445 PMC2865300 20208072

[24] Rauschert N , Brändlein S , Holzinger E , Hensel F , Müller‐Hermelink H‐K , Peter Vollmers H (2008) A new tumor‐specific variant of GRP78 as target for antibody‐based therapy. Lab Investig 4, 375–386. 10.1038/labinvest.2008.2 18268478

[25] Bowman AB and Aschner M (2014) Considerations on manganese (Mn) treatments for *in vitro* studies. Neurotoxicology 41, 141–142. 24509086 10.1016/j.neuro.2014.01.010 PMC4004588

[26] Romani AMP (2011) Cellular magnesium homeostasis. Arch Biochem Biophys 1, 1–23. 10.1016/j.abb.2011.05.010 PMC3133480 21640700

[27] Niesen FH , Berglund H and Vedadi M (2007) The use of differential scanning fluorimetry to detect ligand interactions that promote protein stability. Nat Protoc 2, 2212–2221. 17853878 10.1038/nprot.2007.321

[28] Henderson B and Martin ACR (2014) Protein moonlighting: a new factor in biology and medicine. Biochem Soc Trans 42, 1671–1678. 25399588 10.1042/BST20140273

[29] Carvalho HH , Silva PA , Mendes GC , Brustolini OJB , Pimenta MR , Gouveia BC , Valente MAS , Ramos HJO , Soares‐Ramos JRL and Fontes EPB (2014) The endoplasmic reticulum binding protein BiP displays dual function in modulating cell death events. Plant Physiol 164, 654–670. 24319082 10.1104/pp.113.231928 PMC3912096

[30] Preissler S , Rato C , Chen R , Antrobus R , Ding S , Fearnley IM and Ron D (2015) AMPylation matches BiP activity to client protein load in the endoplasmic reticulum. Elife 4, 1–33. 10.7554/eLife.12621 PMC4739761 26673894

[31] Carlino A , Toledo H , Skaleris D , DeLisio R , Weissbach H and Brot N (1992) Interactions of liver Grp78 and Escherichia coli recombinant Grp78 with ATP: multiple species and disaggregation. Proc Natl Acad Sci USA 89, 2081–2085. 1532251 10.1073/pnas.89.6.2081 PMC48600

[32] Toledo H , Carlino A , Vidal V , Redfield B , Nettleton MY , Kochan JP , Brot N and Weissbach H (1993) Dissociation of glucose‐regulated protein Grp78 and Grp78‐IgE Fc complexes by ATP. Proc Natl Acad Sci USA 90, 2505–2508. 8460165 10.1073/pnas.90.6.2505 PMC46116

[33] Gauts JR and Hendershotsqv LM (1993) Mutations within the nucleotide binding site of immunoglobulin‐ binding protein inhibit ATPase activity and interfere with release of immunoglobulin heavy chain. J Biol Chem 268, 7248–7255. 8463260

[34] Flynn GC , Chappell TG and Rothman JE (1989) Peptide binding and release by proteins implicated as catalysts of protein assembly. Science 245, 385–390. 2756425 10.1126/science.2756425

[35] Romani AMP (2011) Cellular magnesium homeostasis. Arch Biochem Biophys 512, 1–23. 21640700 10.1016/j.abb.2011.05.010 PMC3133480

[36] Schmid SL , Braell WA and Rothman JE (1985) ATP catalyzes the sequestration of clathrin during enzymatic uncoating. J Biol Chem 260, 10057–10062. 2862148

[37] Misselwitz B , Staeck O , Matlack KES and Rapoport TA (1999) Interaction of BiP with the J‐domain of the Sec63p component of the endoplasmic reticulum protein translocation complex. J Biol Chem 274, 20110–20115. 10400622 10.1074/jbc.274.29.20110

[38] Alder NN , Shen Y , Brodsky JL , Hendershot LM and Johnson AE (2005) The molecular mechanisms underlying BiP‐mediated gating of the Sec61 translocon of the endoplasmic reticulum. J Cell Biol 168, 389–400. 15684029 10.1083/jcb.200409174 PMC2171714

[39] Flynn GC , Pohl J , Flocco MT and Rothman JE (1991) Peptide‐binding specificity of molecular chaperone BiP. Nature 353, 726–730. 1834945 10.1038/353726a0

[40] Blond‐Elguindi S , Fourie AM , Sambrook JF and Gething MJH (1993) Peptide‐dependent stimulation of the ATPase activity of the molecular chaperone BiP is the result of conversion of oligomers to active monomers. J Biol Chem 268, 12730–12735. 8509407

